# Degradation and fragmentation behavior of polypropylene and polystyrene in water

**DOI:** 10.1038/s41598-022-23435-y

**Published:** 2022-11-02

**Authors:** Hisayuki Nakatani, Yuina Ohshima, Taishi Uchiyama, Suguru Motokucho

**Affiliations:** Polymeri Materials Laboratory, Chemistry and Materials Program, Nagasaki University, 1-14 Bunkyo-Machi, Nagasaki, 852-8521 Japan

**Keywords:** Environmental chemistry, Materials chemistry, Polymer chemistry, Chemistry

## Abstract

The polystyrene (PS) retrieved from the beach exhibited no change in surface texture. In contrast to it, the retrieved polypropylene (PP) had a rumpled surface texture. Highly reactive sulfate radical generated by K_2_S_2_O_8_ was employed as degradation initiator of PP and PS, and their degradation behavior was studied in water. The PS carbonyl index value gradually went up down, and its molecular weight (MW) curve discontinuously shifted to a lower MW with the increase of the degradation time unlike the PP. It was found that the PP microplastic production rate was approximately three time higher than the PS from weight ratio dependence on degradation time. The higher microplastic production rate of PP arose from its crystallizability. The voids were produced by change in specific volume occurring by chemi-crystallization and then provoked the cracks leading to quick fragmentation. The SEM photographs suggested that the PP microplastic size facilely reached nm order by the cracking around lamella.

## Introduction

Recently accumulation of immense amount of plastic production litter is becoming a big issue in a marine environment^1–11^. The plastic litter has been widespread in the oceans, resulting that it provokes microplastic (MP) pollution^3,4,6,9^. It is well-known that polypropylene (PP), polyethylene (PE) and polystyrene (PS) products are a main source of MP. The MP floats on the sea surface since these polymers are low density. It has been reported that the MP is formed in water by light exposure in the visible and/or UV regions^12–14^. In our previous study, PP degradation tests were performed in water with a specific photocatalyst under visible light irradiation or with an advanced oxidation process (AOP) using sulfate radical^15,16^. The MP particles were obtained by planar exfoliation, i.e. peeling-off^15,16^. From the surface analysis of PP samples retrieved from the two beaches in Japan^16^, it was confirmed that this peeling phenomenon occurred in the sea. Marine MP formation is certainly associated with autoxidation and water. It is considered that there is a certain difference in the MP formation behavior of PP, PE and PS. The autoxidation occurs in the solid state^12–14^. A crystalline polymer such as PP and PE is composed of crystalline and amorphous parts. The complicated matrix certainly affects degradation with autoxidation. In fact, the PP degradation spreading is heterogeneous^17–21^, and its behavior is considerably complicated due to permeability of light, heat, oxygen and diffusibility of degradation initiator^18–21^. On the other hand, PS is an amorphous polymer and is composed of only amorphous part. The degradation spreading behavior is considerably homogeneous. In addition, recrystallization called “chemi-crysallization” is not initiated by the degradation. It is considered that the differences certainly affect mechanism and rate of MP formation. However, a study on MP formation rate and mechanism concerning the difference between crystalline and amorphous polymers has not been performed yet.

In this study, marine PP and expanded PS (EPS) were retrieved to compare surface textures. The texture difference was studied using an optical microscope and FT-IR measurement. Moreover, highly reactive sulfate radical was employed as autoxidation initiator of PP and PS, and their degradation behavior was studied in water to estimate formation rate and to elucidate fragmentation mechanism concerning the difference between crystalline and amorphous polymers.

## Materials and methods

### Materials

PP was supplied by Prime Polymer Co., Ltd. (product name: J-700GP). The MFR and density were 8 g/10 min and 0.9 g/cm^3^. PS was purchased from Sigma-Aldrich Co. LLC. The weight-average molecular weight (Mw) and molecular weight distribution (Mw/Mn) were 3.5 × 10^5^ and 2.1. Potassium hydroxide (KOH), potassium persulfate (K_2_S_2_O_8_) and methanol were purchased from Wako Pure Chemical Industries.

### Marine PP and expanded PS sample collection

Marine PP and expanded PS litter pieces were collected from Head land beach in Chigasaki city, Kanagawa, Japan (see Fig. S1). The detailed location was shown in Fig. S1. A pyrolysis gas chromatography/mass (py-GC/MS: SHIMADZU GCMS-QP2010 PLUS, Japan) analysis was used to discriminate the PP and expanded PS litter pieces from other plastics. These pieces were pretreated using KOH 1 N aqueous solution.

### Degradation using advance oxidation process (AOP)

The PP and PS films were molded into thin films (30 × 30 × 0.075 mm) by compression molding at 180 °C under 10 MPa for 11 min. The AOP degradation procedure was according to reports^22,23^. Each five pieces of the films were put into a100 ml glass vessel equipped with a 20 ml aqueous solution containing 0.54 g K_2_S_2_O_8_ at ca. 65 °C for 12 h under stirring with a stirrer tip speed of ca. 100 rpm, and the equal amount of K_2_S_2_O_8_ aqueous solution was replaced every 12 h due to the consumption of the oxidant. The films were continuously treated according to the above procedures for different time periods. The degradation was performed using the K_2_S_2_O_8_ aqueous solution.

### Weight measurement of PP and PS films degraded by K_2_S_2_O_8_ aqueous solution

The five film pieces at every 3 days were carefully picked up from the glass vessel with tweezers and were rinsed with methanol. After drying with vacuum oven at 60 °C for 7 h, the weight was measured.

### Fourier transform infrared (FT-IR) analysis

The IR spectra were measured with an FT-IR spectrometer (Jasco FT-IR 660 plus) at a resolution of 4 cm^−1^ over the full mid-IR range (500–4000 cm^−1^).

### Gel permeation chromatography (GPC) analysis

Sample was dissolved in 5 ml of *o-*dichlorobenzene or chloroform, and the obtained sample solution was directly measured by GPC. The PP and PS molecular weights were determined by HLC-8321GPC/HT GPC system (Tosoh Co., Ltd.) at 140 °C using *o-*dichlorobenzene and by Prominence GPC system (SHIMADZU Co., Ltd.) at 40 °C using chloroform, respectively.

### Differential scanning calorimetry (DSC) measurement

The DSC measurements were made with a SHIMADZU DSC-60 Plus. The 5 mg samples were sealed in aluminum pans. The measurement of the samples was carried out at a heating rate of 10 °C/min in the measurement range from 30 to 250 °C under a nitrogen atmosphere.

### Scanning electron microscope (SEM) analysis

The SEM analysis was carried out with a JEOL JSM-5800 or JSM-7500FAM with 5.0 kV. The working distance was about 3 × 4 mm. Samples placed in dried oven maintained at 27 °C for 30 min and were sputter-coated with gold before SEM imaging.

## Results and discussion

### Retried PP and PS sample textures

**Figure 1:**
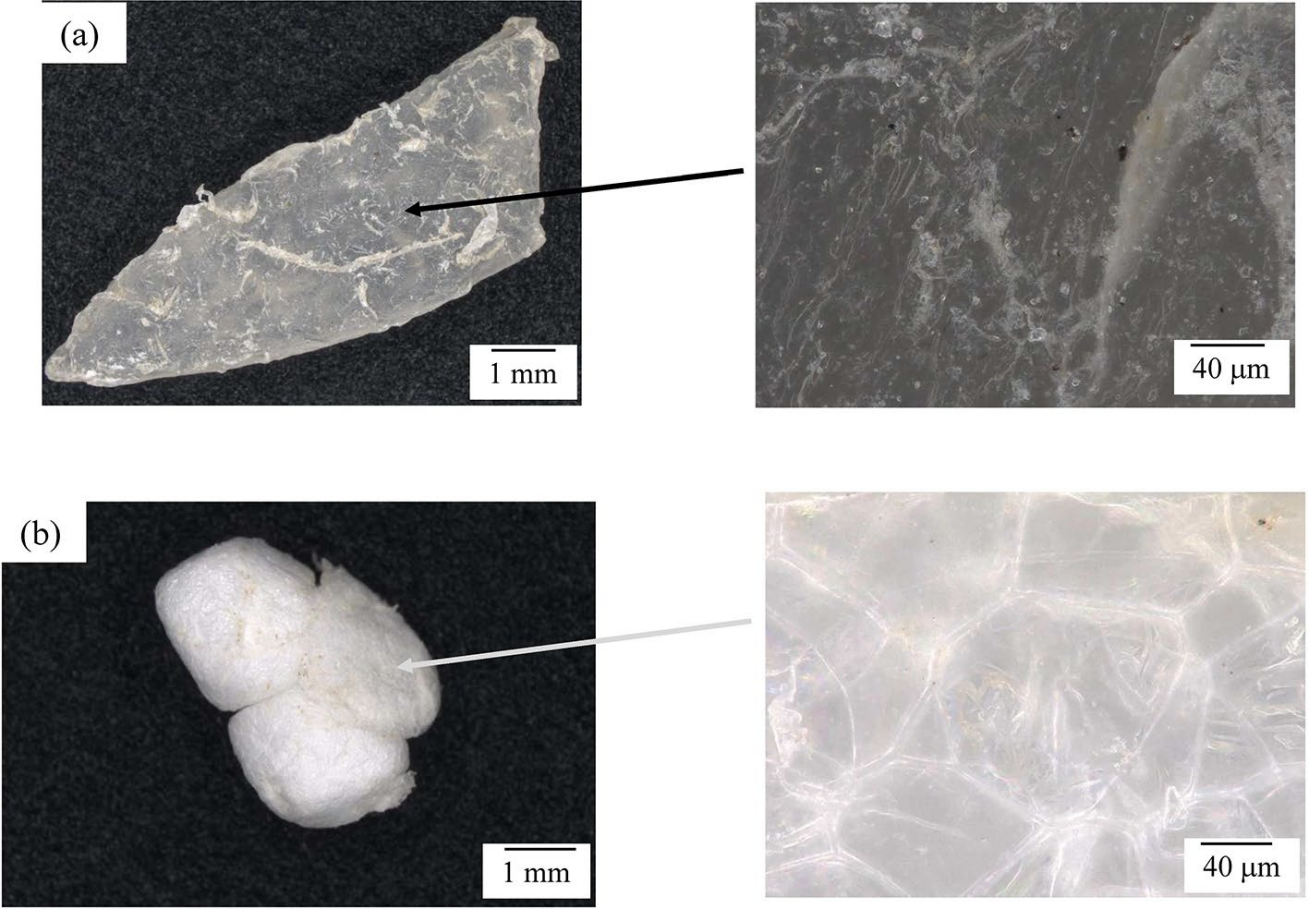
Optical microphotographs of PP and expanded PS (EPS) samples retrieved from beaches on the Sagami bay: (**a**) PP. (**b**) PS.

Figure 1 shows the optical microphotographs of PP and expanded PS (EPS) samples retrieved from beaches on the Sagami bay (see Fig. S1). The PP wrinkles a surface, implying that photodegradation occurs under sunshine irradiation as well as polyethylene^24,25^. The rumpled surface texture is generated by autoxidation accompanying polymer chain scission and carbonyl group production^16^. On the other hand, the EPS keeps cell-like structure without exhibiting the wrinkle texture such as the PP. As shown in Fig. S2, hydroperoxide (–OH) and carbonyl group peaks corresponding to degraded PS can be distinctly observed in these spectra of three EPS samples retrieved from the beach on the Sagami bay^26^. We consider that an effect of PS degradation in the sea (water) on the surface texture is smaller than that of PP. The PS oxidative reactivity is almost the same because of the polymers having tertiary hydrocarbon groups in the polymer chains. The difference of texture change would be associated with degradation spread. It is well-known that PS degradation is initiated by autoxidation as well as PP^27,28^. However, the spread behavior of degradation is considerably different due to scission position of polymer chain and presence of crystalline part. Moreover, water presence provokes fragmentation, resulting that the degradation behavior is complicated^16^. To clarify degradation behavior of PP and PS in the sea, it is necessary to perform autoxidation in water.

### AOP degradation in water

**Figure 2:**
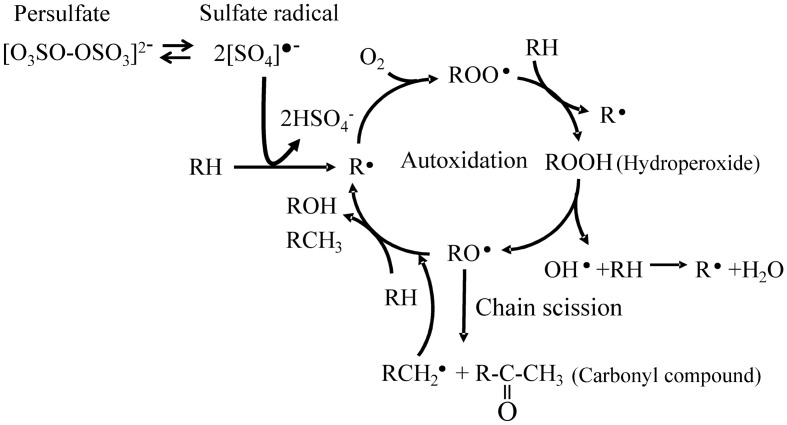
Autoxidation mechanism initiated by advanced oxidation process (AOP).

**Figure 3:**
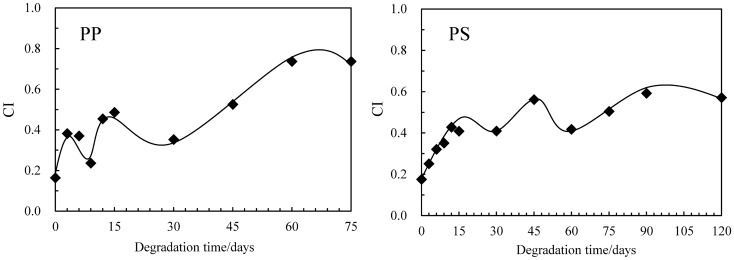
Value of carbonyl index (CI) change vs degradation time.

**Figure 4:**
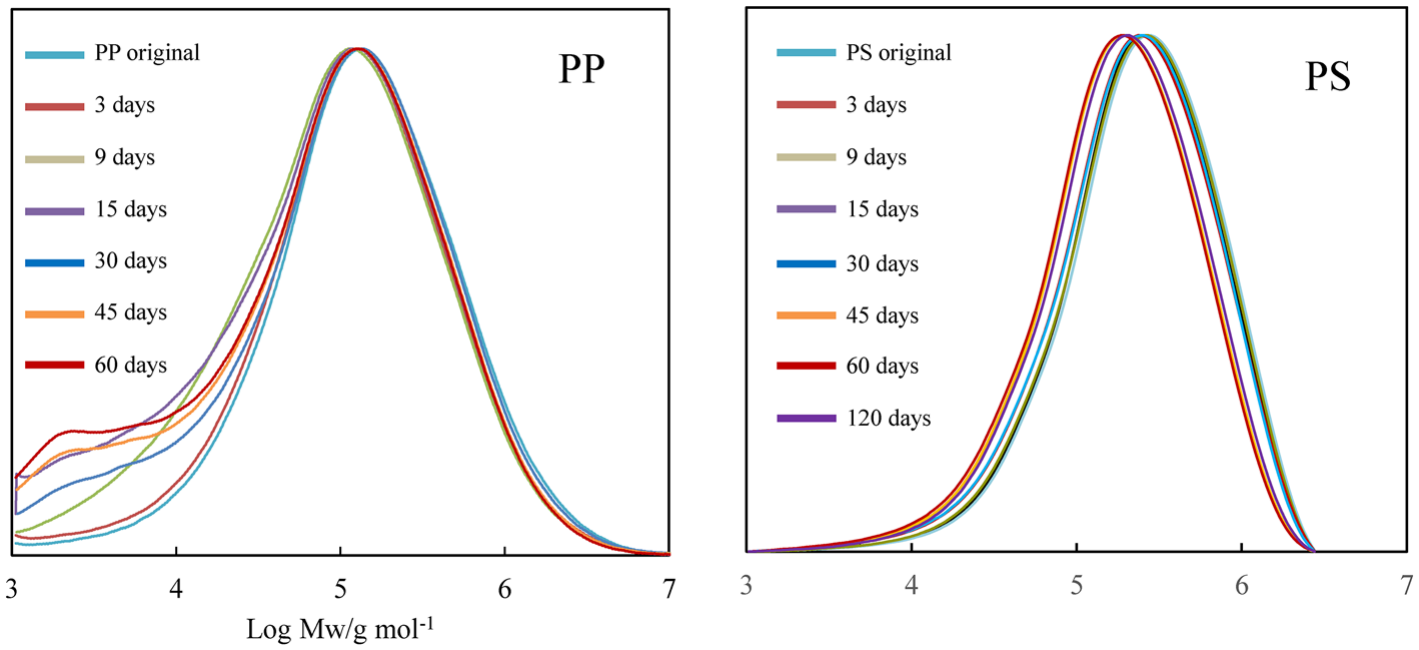
PP and PS differential molecular weight distribution curve changes vs degradation time.

**Figure 5:**
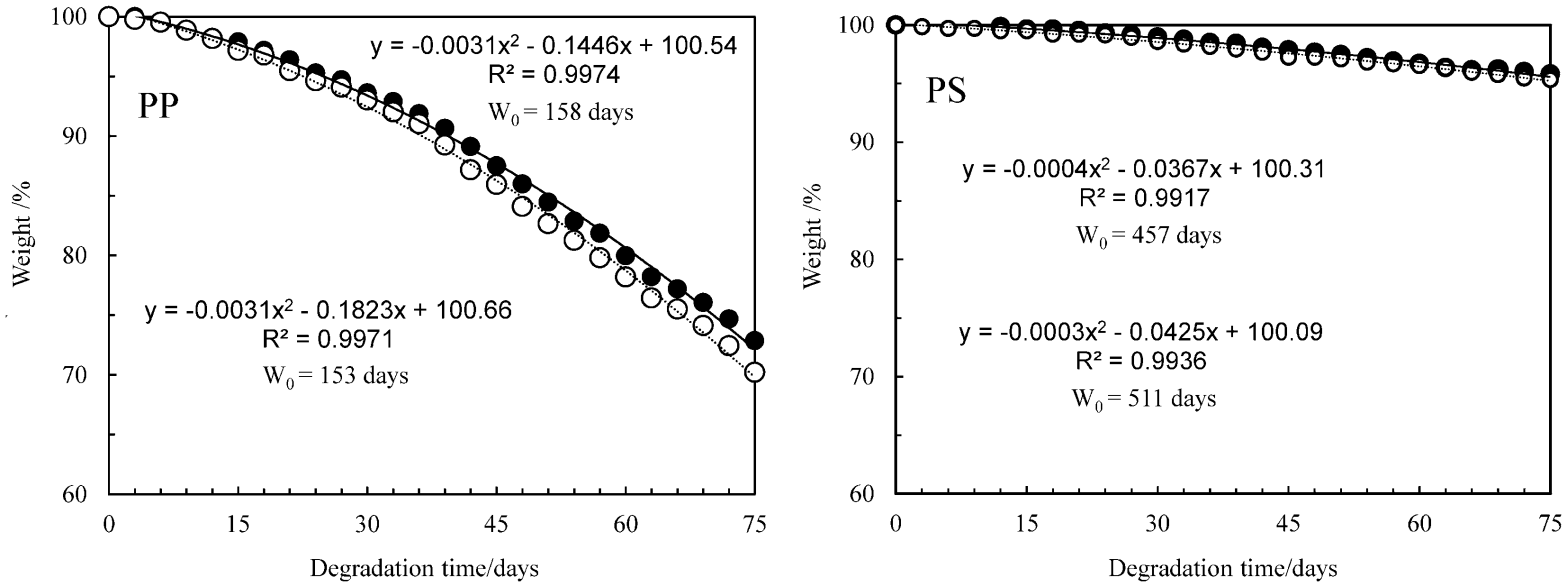
PP and PS weight ratio vs degradation time.

The initiation rate of autoxidation is considerably slow under sunlight irradiation. Therefore, K_2_S_2_O_8_ was employed as the initiator^22,23^. Highly reactive sulfate radicals are generated by cleaving the peroxide bond in K_2_S_2_O_8_ and are attacking C–C polymer chain as shown in Fig. 2. Figure 3 shows the PP and PS carbonyl index (CI) values vs degradation time. The CI of PP was calculated using the carbonyl group / scissoring CH_2_ group band intensity ratio^29^, and that of PS was done using the carbonyl group / CH_2_ stretching band intensity ratio^30^. Both of the CI values go up down. The behavior is attributed to the repeated oxidation and peeling off^15,16^. In particular, the CI of PP distinctly exhibits up and down values against the degradation time. Although the similar oscillation can be seen in the PS, the amplitude and period are considerably smaller and longer than those of PP. These behavior implies difference between PP and PS degradation in water. Figure 4 shows the PP and PS differential molecular weight (MW) distribution curve changes vs degradation time. The peak position of PP MW curve is little changed regardless of increments of degradation time. A small peak at the low MW (ca. 3000) appears instead of the main peak position unchange. The degradation is confined on the PP surface, and inner part is little degraded. The heterogeneous degradation accounts for the distinctive behavior of MW curve change. On the other hand, the MW curves of PS gradually shift to lower MW with the increase of degradation time. The PS degradation is homogeneously progressing as compared with the PP. Figure 5 shows the PP and PS weight ratio vs degradation time in water. The weight change with the degradation progress is related to the chemical and physical factors by autoxidation and by detaching form the polymer body, respectively. It seems that the weight of PP or PS and the time satisfy a quadratic polynomial relationship during degradation. Hence we fitted the experimental data using a quadratic polynomial and extrapolate to obtain the period required for completely disappearing. The weight ratio dependence test has been performed twice in each of the PP and PS samples to confirm the reproducibility. As shown in Fig. 5, the test shows good reproducibility and reveals the fragmentation rate, i.e. microplastic production rate. The PP and PS weight ratios drop until about 70% and 95% for the 75 degradation days, respectively. The results suggest that the PP microplastic production rate is higher than PS. The estimation period required for completely disappearing (W_0_) was calculated by polynomial approximation equation. The W_0_ values of PP are 158 and 153 degradation days, and those of PS are 457 and 511 degradation days. The W_0_ value differences reveal that PP microplastic production rate is approximately three time higher than PS. Figure S3 shows the degradation mechanism of PP and PS in AOP progress. The degradation is mainly caused by the chain scission, which is a step in autoxidation cycle reaction. There are no differences between the chain scission mechanisms of PP and PS. Hence the difference in the microplastic production rate is considered to be related to crystallizability of PP chain.

### Chemi-crystallization

**Figure 6:**
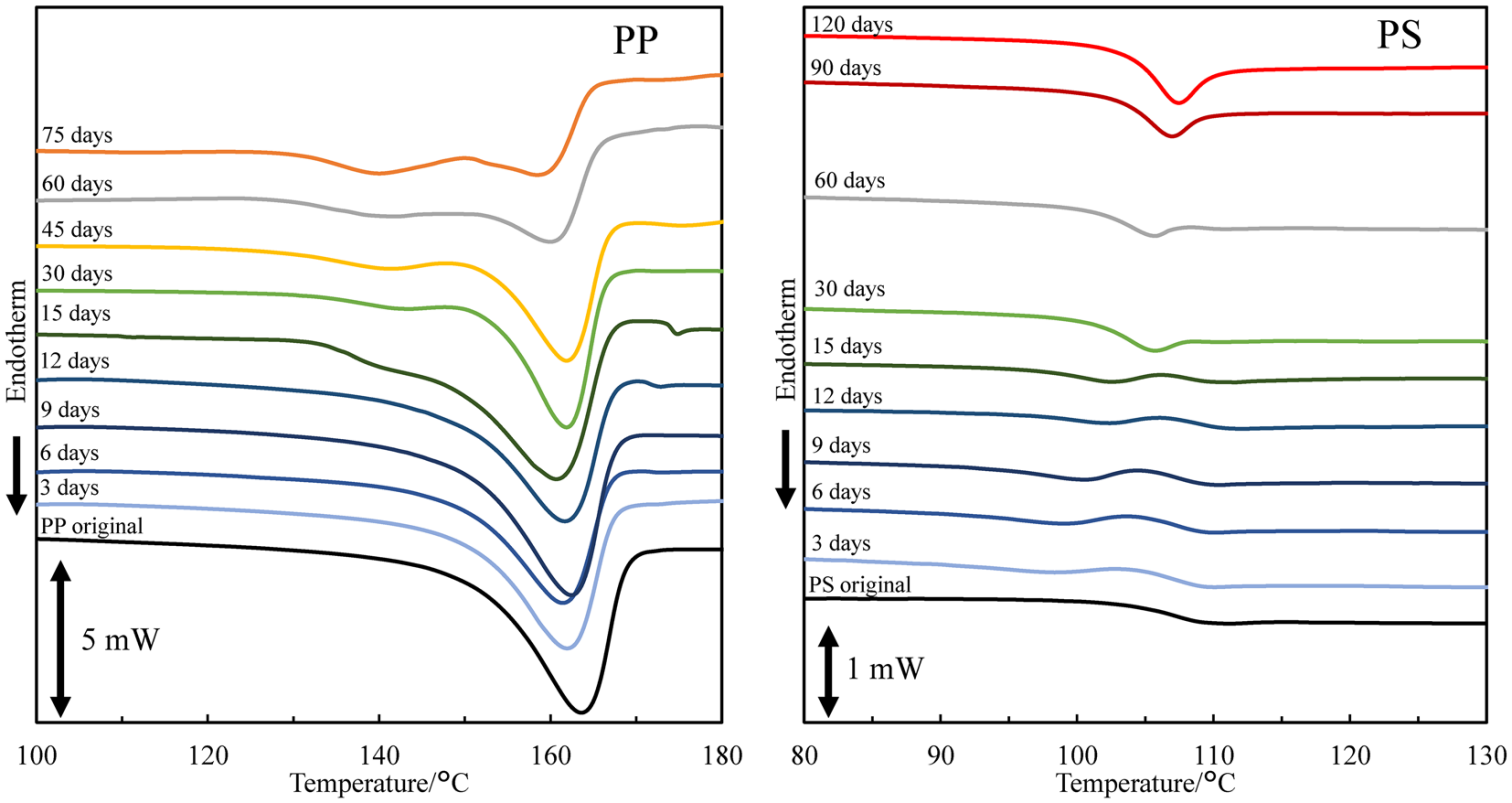
DSC curves of PP and PS samples with various degradation time.

**Figure 7:**
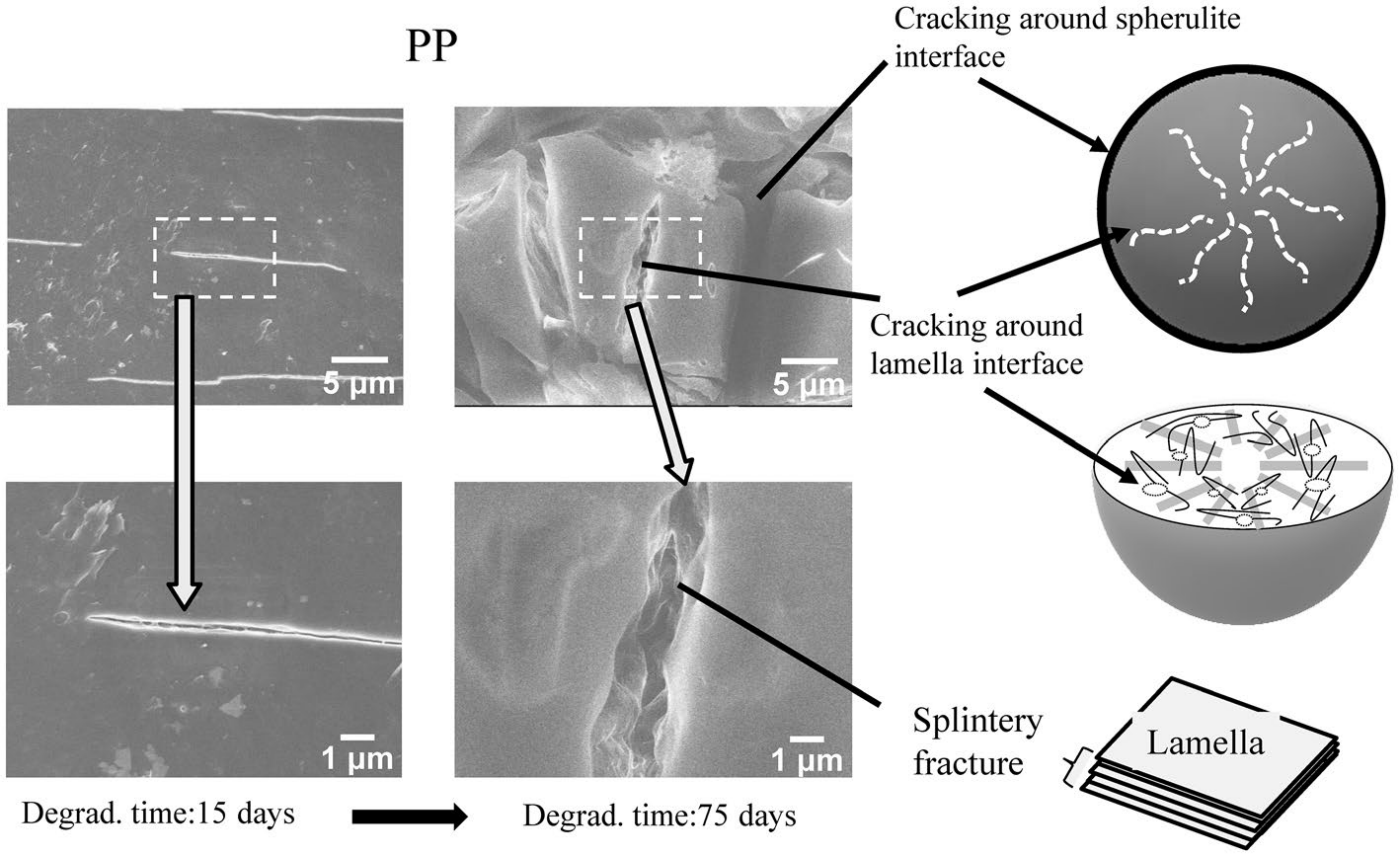
SEM photographs and cracking locations of PP in degradation.

**Figure 8:**
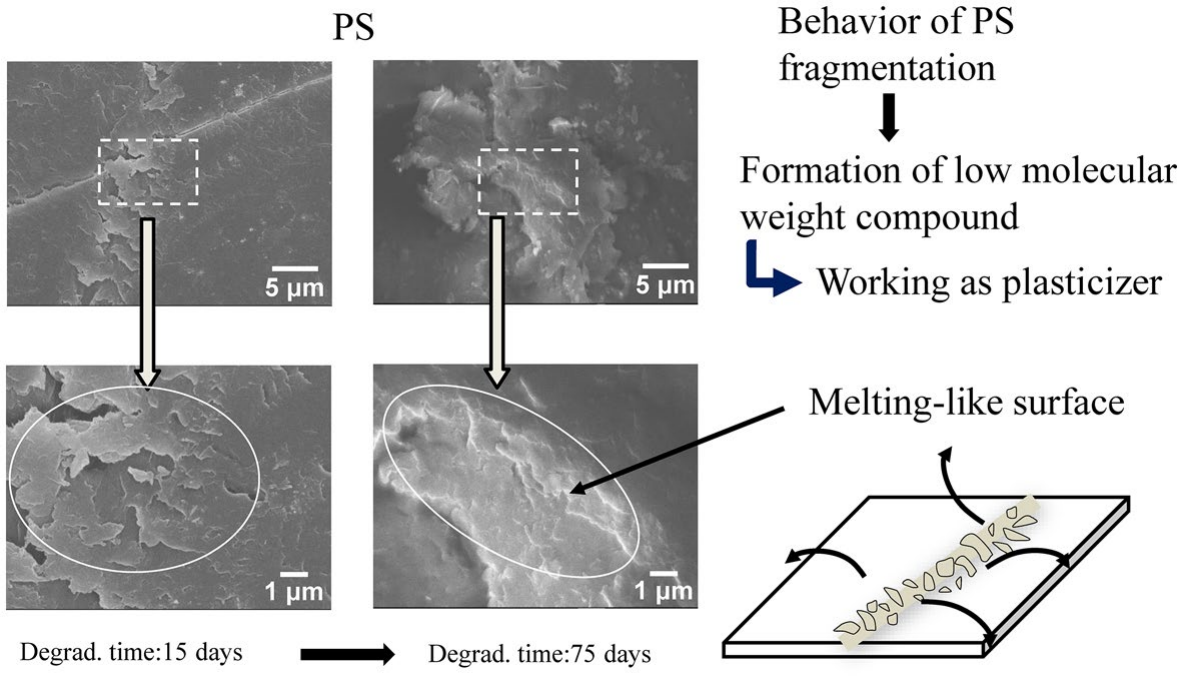
SEM photographs and fragmentation mechanism of PS in degradation.

The higher microplastic production rate of PP would arise from its crystallizability. Figure 6 shows the DSC curves of PP samples with various degradation time. The melting point (Tm) peak shifts to lower temperatures. Moreover, the side Tm peak around 140 °C appears after the 15 degradation days. The Tm change and side Tm birth are typical behavior derived from chemi-crystallization^31,32^. On the other hand, the PS samples do not exhibit such behavior because of amorphous polymer, and characteristic change in the glass transition temperature (Tg) is observed instead of chemi-crystallization. The Tg is 105 °C in the pristine PS sample, and another Tg appears at around 100 °C from the 3 to 15 degradation days. And then the PS samples have only one Tg at 103 °C until the 120 degradation days. The decrease of MW accounts for such Tg behavior because it corresponds to the discontinuous lowering. Figure S4 shows the SEM photographs of PP and PS fibrils in cracks initiated by the degradation. The various width of fibril can be seen in the PP sample, suggesting that the cracks are non-uniformly generated. In contrast to the PP fibril, the PS sample exhibits similar sized fibrils. In addition, some melting fibrils are observed. Crystallizability accounts for the difference between the fibril shapes. In the case of PP, chemi-crystallization is provoked by its crystallizability in the degradation and generates non-uniform cracks leading to birth of fibrils with various widths. Whereas, non-crystallizable PS is plasticized by its lower molecular compound produced in the degradation. The degradation spreading becomes more homogeneous due to plasticizing. The melting PS fibril exhibits that plasticizer exists in the PS degradation. The PS degradation homogeneously proceeds and produces similar sized fibrils. Figure 7 shows the SEM photographs and cracking locations of PP in the degradation. A void is produced by change in specific volume occurring when PP chemi-crystallizes^32–34^. The void becomes a starting point of crack. The 75 days-degraded PP sample exhibits the fine cracks around the spherulite and lamella interfaces. The splintery fracture can be seen and reveals the chemi-crystallization occurring at the lamella interface. The PP fragment size certainly reaches nm order by the cracking around lamella. The chemi-crystallization accounts for higher microplastic production rate. Figure 8 shows the SEM photographs and fragmentation mechanism of PS in the degradation. A mark of scaly peeling off can be seen on the 15 days-degraded PS sample, and then melting-like surface is observed at the 75 degradation days. The low molecular compound produced by the PS degradation is concerned with such PS fragmentation behavior. It certainly works as plasticizer, and rate of peeling off slows down. The plasticizer work accounts for the lower microplastic production rate.

## Conclusion

The effect of PS degradation in the sea on the surface texture was smaller than that of PP. The difference of texture change was associated with crystallizability. Highly reactive sulfate radical generated by K_2_S_2_O_8_ was employed as the degradation initiator. The CI value of PS gradually went up down with the increase of the degradation time as compared with that of PP. The characteristic behavior of PP MW curve revealed that the degradation was confined on the PP surface. On the other hand, the MW curves of PS gradually shifted to lower MW with the increase of degradation time, resulting that the degradation was homogeneously progressing. The PP DSC showed that chemi-crystallization occurred in the degradation. It was found that the higher microplastic production rate of PP arose from its crystallizability. The void was produced by change in specific volume occurring by the chemi-crystallization and then provoked the cracks leading to quick fragmentation. PP fragment size certainly reached nm order by the cracking around lamella. The chemi-crystallization accounted for higher microplastic production rate. In the case of PS fragmentation, the low molecular fraction produced by the degradation worked as plasticizer, and its fragmentation rate slowed down. The plasticizer work accounts for the lower microplastic production rate.

## Supplementary Information

Supplementary Legends.

Supplementary Figure S1.

Supplementary Figure S2.

Supplementary Figure S3.

Supplementary Figure S4.

## Data Availability

The datasets generated and/or analyzed during the current study are available from the corresponding author on reasonable request.
