# Author Correction: Degradation and fragmentation behavior of polypropylene and polystyrene in water

**DOI:** 10.1038/s41598-022-26982-6

**Published:** 2022-12-29

**Authors:** Hisayuki Nakatani, Yuina Ohshima, Taishi Uchiyama, Suguru Motokucho

**Affiliations:** Polymeri Materials Laboratory, Chemistry and Materials Program, Nagasaki University, 1‑14 Bunkyo‑Machi, Nagasaki, 852‑8521 Japan

Correction to: Scientific Reports https://doi.org/10.1038/s41598-022-23435-y, Published online 02 November 2022.

The original version of this Article contained an error in the spelling of the author Suguru Motokucho which was incorrectly given as Motokucho Suguru.

The original Article has been corrected.

