# Supplementary material for: Degradation and fragmentation behavior of polypropylene and polystyrene in water
Source: Sci Rep. 2022 Nov 2;12:18501. doi: 10.1038/s41598-022-23435-y (PMC9630436; doi:10.1038/s41598-022-23435-y)
Supplement: Supplementary file 1 — Supplementary Legends. [file 41598_2022_23435_MOESM1_ESM.docx]

Supplement captions

Figure S1 Sampling locations of PP and expanded PS (EPS) samples. Local names and other information are given in Text.

Figure S2 FT-IR spectra of three EPS samples retrieved from beaches on the Sagami bay.

Figure S3 Degradation mechanism of PP and PS in AOP progress.

Figure S4 SEM photographs of PP and PS fibrils in cracks initiated by degradation.
