# Supplementary figures and images for: Degradation and fragmentation behavior of polypropylene and polystyrene in water
Source: Sci Rep. 2022 Nov 2;12:18501. doi: 10.1038/s41598-022-23435-y (PMC9630436; doi:10.1038/s41598-022-23435-y)

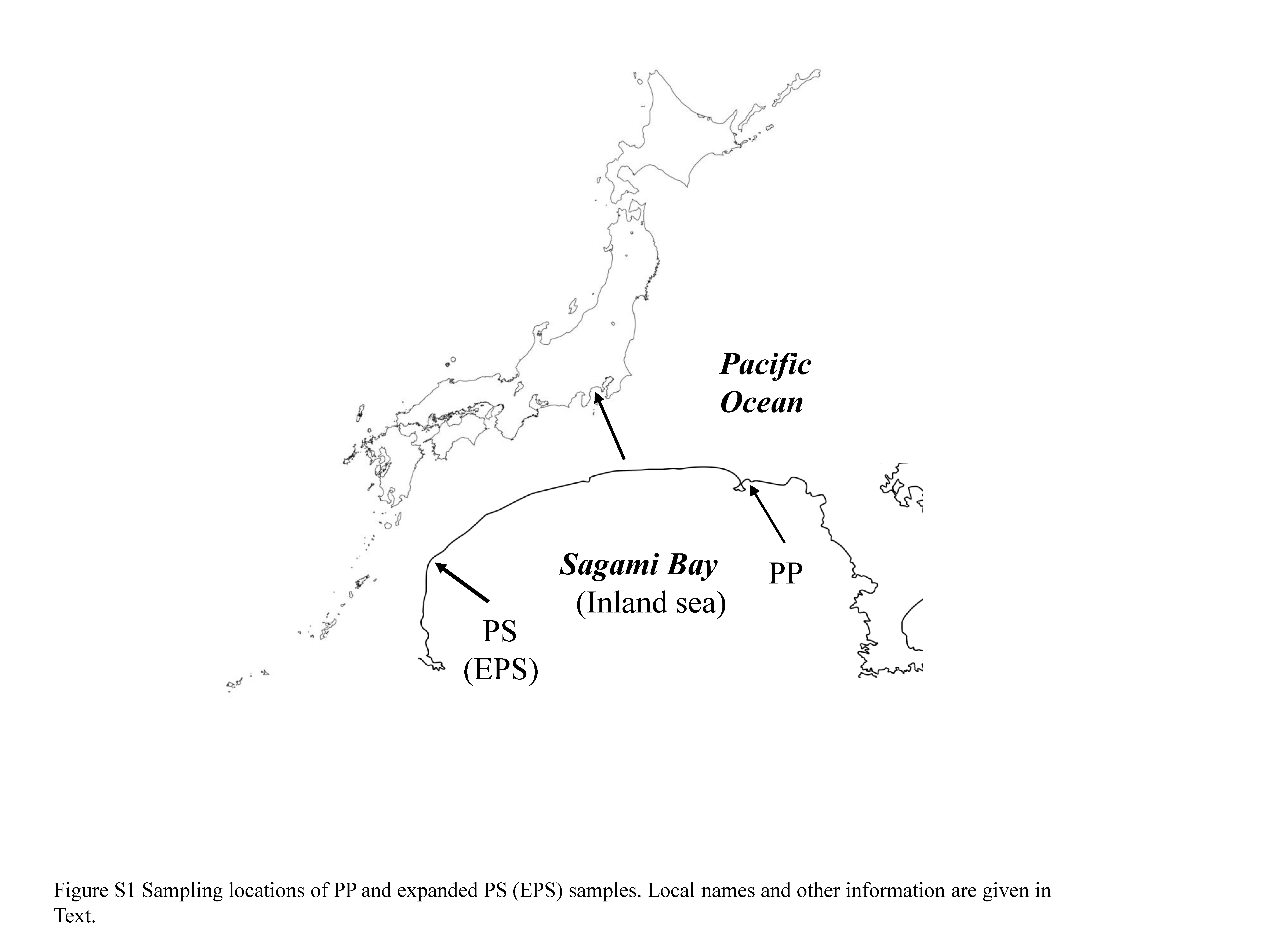

Supplement: Supplementary file 2 — Supplementary Figure S1. [file 41598_2022_23435_MOESM2_ESM.tiff]

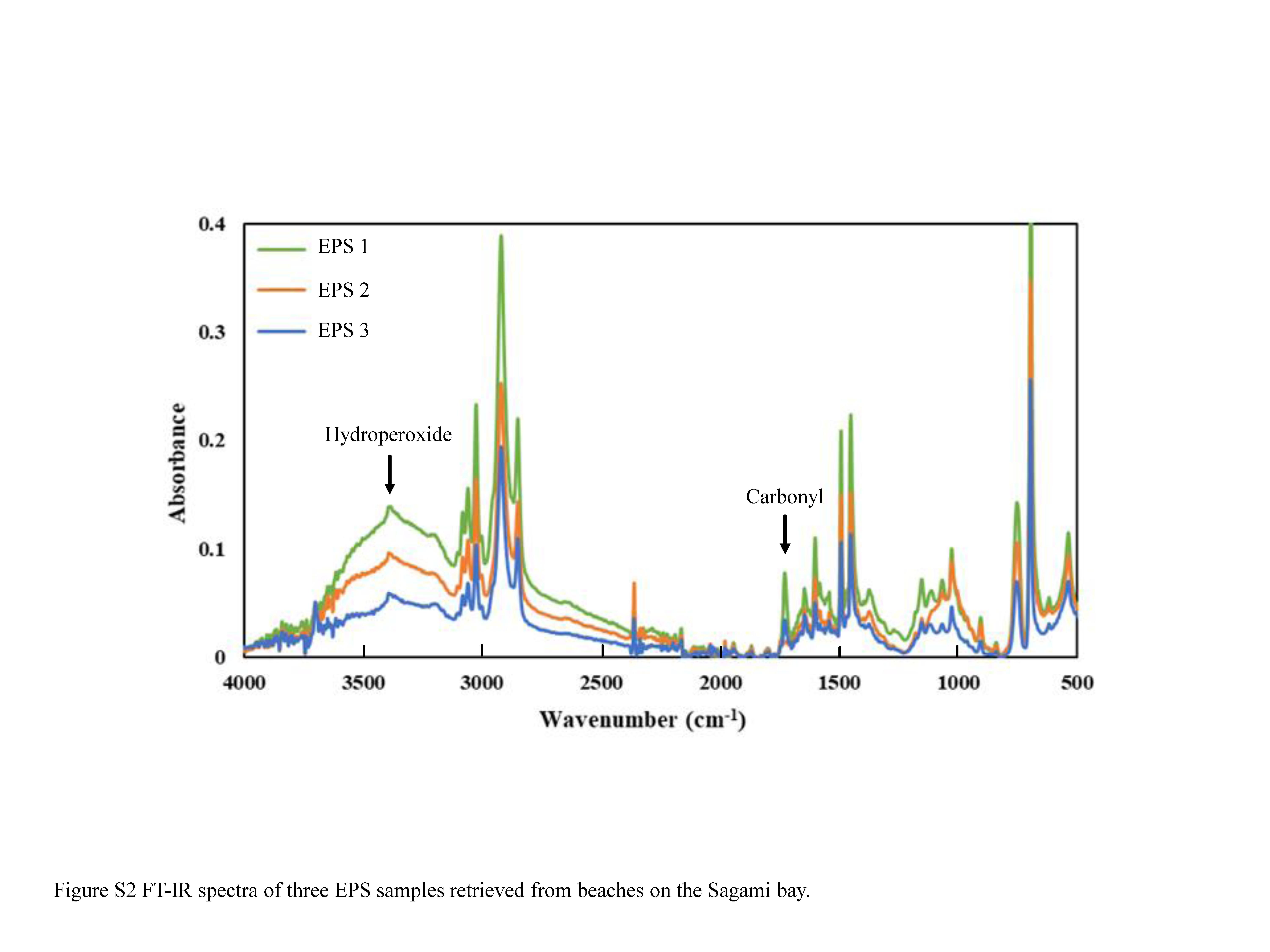

Supplement: Supplementary file 3 — Supplementary Figure S2. [file 41598_2022_23435_MOESM3_ESM.tiff]

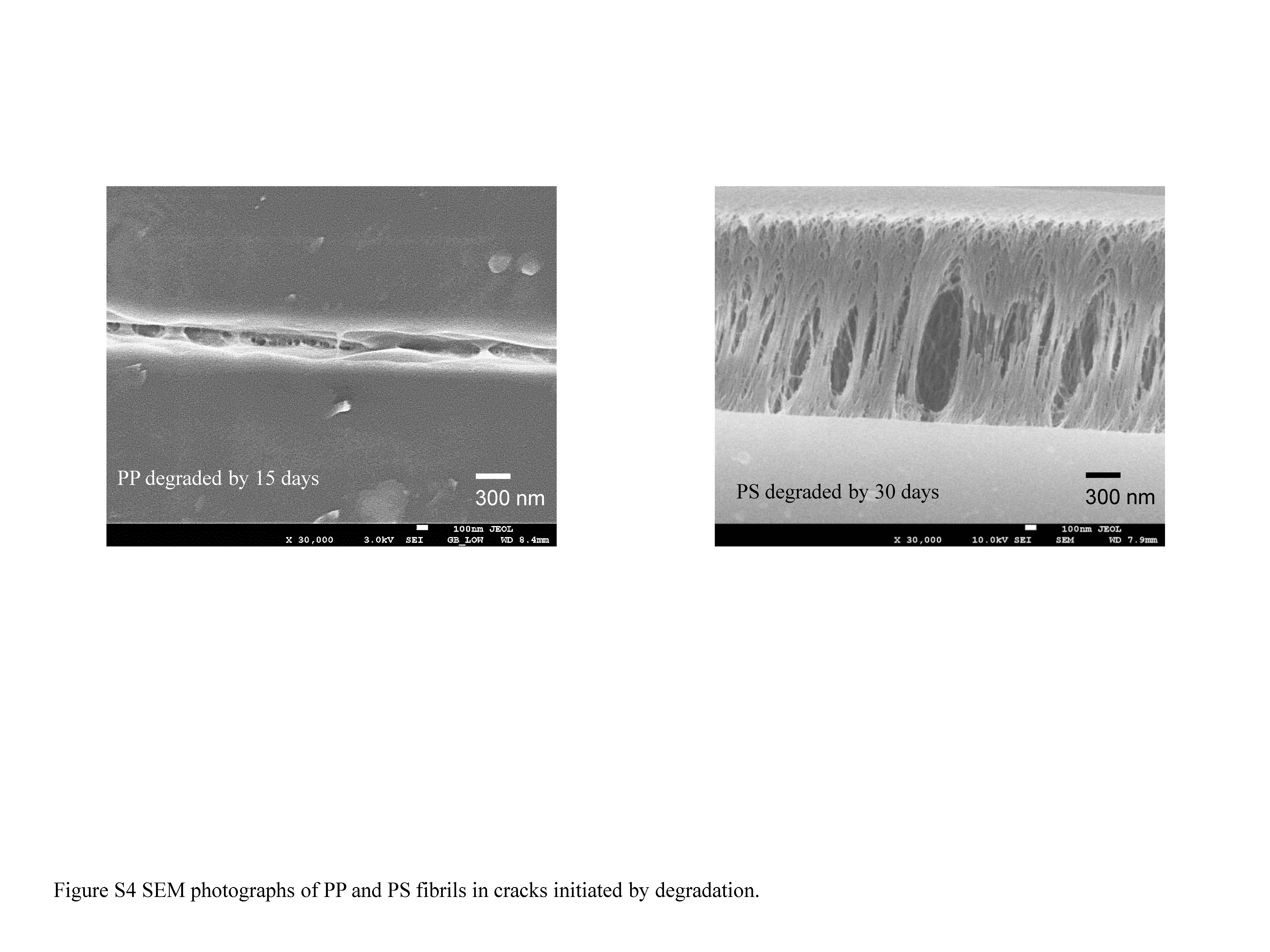

Supplement: Supplementary file 5 — Supplementary Figure S4. [file 41598_2022_23435_MOESM5_ESM.tiff]
